# Dynamic Profiling of Antitumor Activity of CAR T Cells Using Micropatterned Tumor Arrays

**DOI:** 10.1002/advs.201901829

**Published:** 2019-09-30

**Authors:** Xiao Wang, Irene Scarfò, Andrea Schmidts, Mehmet Toner, Marcela V. Maus, Daniel Irimia

**Affiliations:** ^1^ BioMEMS Resource Center Department of Surgery Massachusetts General Hospital Boston MA 02114 USA; ^2^ Shiners Hospitals for Children Boston MA 02114 USA; ^3^ Harvard Medical School Boston MA 02115 USA; ^4^ Cellular Immunotherapy Program Massachusetts General Hospital Cancer Center Charlestown MA 02129 USA; ^5^ Broad Institute of Harvard and MIT Cambridge MA 02142 USA

**Keywords:** cancer immunotherapy, CAR T cells, high‐content screening technology, micropatterned cell array

## Abstract

Cancer immunotherapy based on the engineering of chimeric antigen receptors (CAR) on T cells has emerged as one of the most promising new therapies for patients with B‐cell malignancies. Preclinical assessments of essential CAR T cell functions such as trafficking and cytotoxicity are critical for accelerating the development of highly effective therapeutic candidates. However, current tools for evaluating CAR‐T functions lack sufficient precision. Here, a micropatterned tumor array (MiTA) is described that enables detailed and dynamic characterization of CAR T cell trafficking toward tumor‐cell islands and subsequent killing of tumor cells. It is shown that CAR T cells often merge into large clusters that envelop and kill the tumor cells with high efficiency. Significant differences are also measured between CAR T cells from different donors and between various CAR T cell constructs. Overall, the assay allows for multifaceted, dynamic, high‐content evaluation of CAR T trafficking, clustering, and killing and could eventually become a useful tool for immune‐oncology research and preclinical assessments of cell‐based immunotherapies.

## Graphical abstract

**A micropatterned tumor array enables multifaceted and dynamic characterization of antitumor activity of CAR T cells**. The assay demonstrates that CAR T cells migrate and merge into clusters that envelop the tumor islands and eliminate the tumor cells. It reveals significant differences in antitumor function between CAR T cells from different donors and between different CAR T cell constructs.

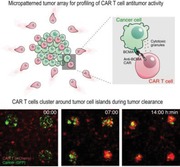

## 1 Introduction

Chimeric antigen receptors (CARs) are engineered receptors used to reprogram patient's T cells to specifically target tumor cells. Cancer immunotherapy based on CAR T cells has emerged as one of the most promising new therapies for the treatment of patients with B‐cell malignancies.^1^, ^2^, ^3^, ^4^, ^5^, ^6^, ^7^, ^8^ The antitumor activity of CAR T cells relies on efficient CAR T cell trafficking to cancer niches, recognition of tumor antigen, and potent cytotoxicity toward tumor cells. These biological processes are dynamic and involve collective interactions of CAR T and tumor cells. Comprehensive preclinical assessments of these key processes are of pivotal importance for ensuring CAR T therapeutic efficacy.

Several in vitro assays can measure CAR T cell cytotoxicity toward tumor cells. Chromium (Cr‐51) release assay is the gold standard for quantifying cytotoxicity in the study of tumor cytolysis.^9^ However, this assay only provides simple end‐point readouts and does not distinguish the CAR T‐mediated killing of target cells from other causes of target cell death. Also, the assay is cumbersome to implement and poses safety challenges because it involves the use of radioactive materials. Assays based on the quantification of cytosolic enzymes such as lactose dehydrogenase (LDH)^10^ or glyceraldehyde phosphate dehydrogenase (GAPDH)^11^ circumvent the need for radioactive materials. However, these assays fail to distinguish the death of target cells from effector cells, since both release cellular enzymes upon lysis. This problem is avoided when firefly luciferase (Fluc) ‐expressing cells are employed as targets of effector T cells and the release of Fluc into the medium is a specific measure of target cell lysis. However, despite being widely used, these biochemical assays are limited to quantifying only the bulk responses at a single time point which can hardly elucidate the complex and dynamic antitumor activity of CAR T cells.

The integration of real‐time monitoring techniques such as time‐lapse imaging^12^ and electrical impedance sensors^13^ with cell culture plates have enabled the dynamic characterization of the cytolysis process. However, the random arrangement of cells in traditional cell culture dishes prohibits the study of CAR T cell trafficking. Lab‐on‐a‐chip technology such as microfluidic cell culture and organ‐on‐chips hold great promise for advancing the therapeutic screening of cancer immunotherapies.^14^, ^15^, ^16^, ^17^, ^18^ However, they are labor‐intensive and difficult to use.^19^ Reproducing sophisticated in vitro microenvironment usually takes days^18^, ^19^, ^20^ and the complexity of the microfluidic systems adversely affect the robustness of measurements.^19^

To overcome the challenges of aforementioned approaches, we designed an assay for the dynamic profiling of antitumor activity of CAR T cells. We employed micropatterning to precisely pattern multiple myeloma tumor cells into arrays of microscale islands.^21^ The tumor‐cell islands have uniform size and shape and contain a similar number of tumor cells, facilitating the reproducibility of screening. Spatially segregating the tumor cells into microscale islands toward which CAR T cells have to actively migrate allows the systematic study of trafficking and subsequent tumor killing. The arrays of tumor‐cell islands are housed in customized microfluidic chambers which eliminates the drifting of CAR T cells caused by convection‐induced flow, ensuring robust cell interactions. We found that CAR T cells robustly migrate toward the tumor‐cell islands, increasing the local effector‐cell density and aggregating into large clusters that envelop the tumor cells and exert cytolytic effects on the tumor cells. The assay detects and quantifies differences in trafficking, clustering, and cytotoxicity of CAR T cells from different donors. Using the assay, we conducted multifaceted characterizations of anti‐BCMA and APRIL‐based CAR T cell constructs against BCMA+ and BCMA knockout (KO) multiple myeloma MM.1s tumor cells. We demonstrate that APRIL‐based CAR T cells efficiently clustered around and eliminated both tumor‐cell types, suggesting that this CAR T cell construct could reduce the incidence of antigen‐negative escape. With the ease of use, the high throughput and reproducibility and the ability to dynamically map the antitumor activity of CAR T cells, the micropatterned tumor array (MiTA) could become a useful tool for studying cancer immunology and aiding the preclinical evaluations of cell‐based cancer immunotherapy.

## 2 Results

### 2.1 Micropatterned Tumor Arrays

**Figure 1:**
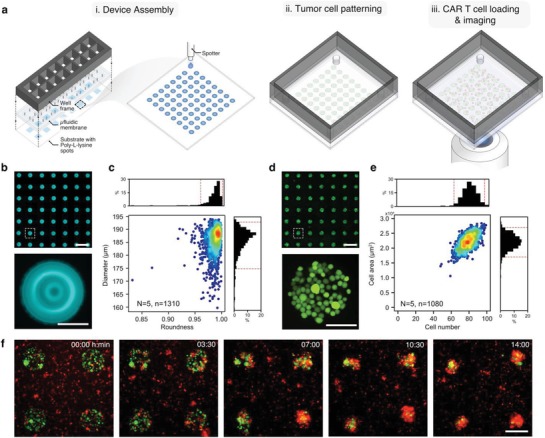
Micropatterned tumor arrays (MiTA) for the quantification of CAR T cell killing. a) Schematic illustrations showing the assembly of the 16 well device, zoom‐in of one well during the printing of the 64 spots, tumor‐cell patterning in the wells, and subsequent CAR T cell loading and imaging. b) Fluorescent microscopic images of a poly‐L‐lysine spot array printed with the automated liquid dispenser and a zoom‐in picture of one spot. The image is pseudocolored cyan. The scale bars in the top and bottom panels are 500 and 100 µm, respectively. c) A heat‐scattered plot of the diameter versus roundness of spots. The top and side histograms correspond to the roundness and the diameter, respectively. The red dashed lines indicate the 95% range. *N* = 1310 islands, *N* = 5 experimental repeats. d) A fluorescent microscopic image showing an array of tumor‐cell islands after patterning. The bottom panel is a zoom‐in picture of the region indicated with a white dashed square. The image is pseudocolored green. The scale bars in the left and right panels are 500 and 100 µm, respectively. *N* = 1080 islands, *N* = 5 repeats. e) A heat‐scattered plot of tumor‐cell number versus cell island area. The top and side histograms correspond to cell number and cell island area, respectively. The red dashed lines indicate the 95% range. f) Time‐lapse fluorescent microscopic images show anti‐BCMA CAR T cells (red) and BCMA tumor‐cell islands (green). The CAR T cells start initially uniformly distributed and end concentrated on top of the tumor‐cell islands after 14 h. The scale bar is 200 µm.

We patterned a large array of microscale tumor‐cell islands that are housed in a microfluidic compartment. First, an array of 1024 spots of an adhesion‐promoting material that consists of a mixture of poly‐L‐lysine and ZETAG were patterned on 1 × 3” glass substrates using an automated liquid dispenser (**Figure** **1**a, panel i). The patterned adhesive spots have a uniform diameter of 185.5 ± 5.2 µm and an average roundness index of 0.99 ± 0.01. Then, we assembled on top of the patterned glass substrate, a polydimethylsiloxane (PDMS) membrane containing 16 microfluidic chambers and a plastic frame defining 16 wells (Figure 1a, panel i). After assembly, the substrate is divided into 16 individual compartments. Each compartment contains 64 spots spaced 500 µm apart, in an 8 × 8 array (Figure 1a, zoom‐in in panel i).

To form the tumor‐cell islands, we load a suspension of tumor cells inside the microfluidic chambers and allow them to sediment and adhere on the spots. We then remove the nonadhered cells by gentle wash (Figure 1a, panel ii). Patterning Roswell Park Memorial Institute growth medium (RPMI) 8226 tumor cells yields spot arrays with an average 79 ± 7 cells per spot and an average 2.2 ± 0.2 × 10^4^ µm^2^ area (Figure 1d,e). The variation of the cell number and area between spots stems from the heterogeneity of the cell size. Finally, we load CAR T cells inside the microfluidic compartments and allow them to sediment. Immediately, we start monitoring the interactions between CAR‐T and tumor cells using time‐lapse imaging (Figure 1a, panel iii). With the assay, we are capable of quantifying the dynamic interactions between CAR‐T and tumor cells (Figure 1f) on 4096 spots on four slides, in 64 different conditions in each experiment (Figure S1, Supporting Information).

### 2.2 Endpoint Evaluation of Overall CAR‐T Antitumor Efficacy Using MiTA

**Figure 2:**
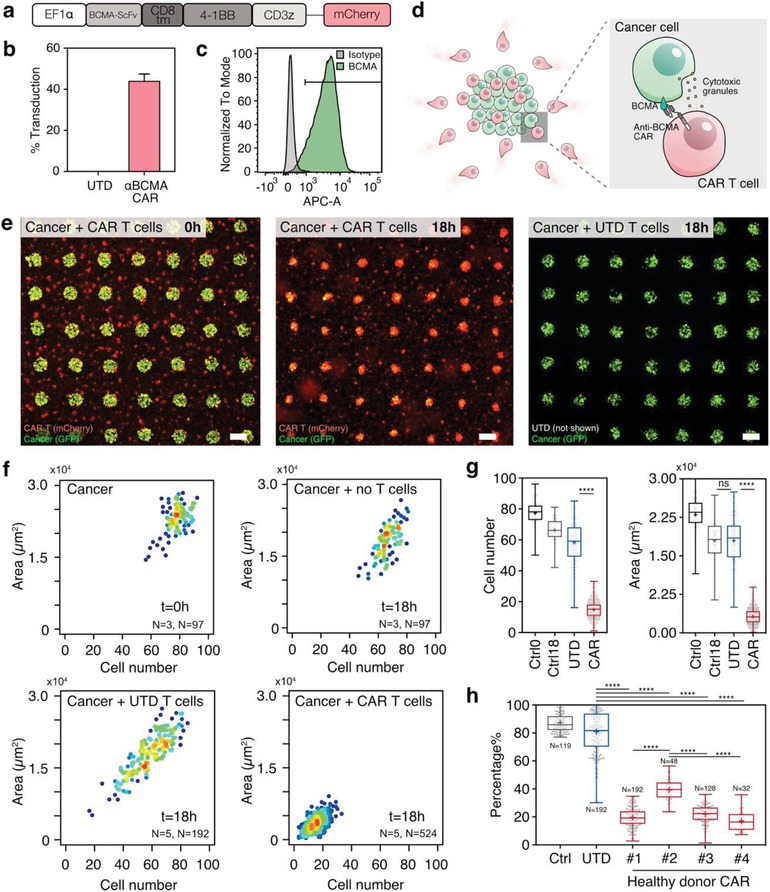
Endpoint evaluation of overall CAR‐T antitumor efficacy using MiTA. a) Schematics of second‐generation anti‐BCMA chimeric antigen receptor construct. b) Expanded T cells from healthy donors included variable anti‐BCMA CAR expression with mean transduction of 44%. (*N* = 3 donors, bars represent SEM). c) FACS plot of RPMI 8226 multiple myeloma cell line stained with anti‐BCMA APC antibody or isotype control. d) BCMA–anti‐BCMA–CAR interactions mediate tumor cells (green) recognition and killing by CAR T cells (red). e) Fluorescent microscopic images showing the snapshots of the interaction of CAR T cells (red) and tumor cell islands (green) at i) 0 and ii) 18 h. In control experiments, UTD T cells had limited interaction with tumor cells after 18 h. The scale bars are 200 µm. f) Heat‐density scatter plots of tumor‐cell island area versus tumor‐cell number for tumor cells alone at 0 and 18 h, tumor cells with UTD T cells at 18 h, and tumors with CAR T cells at 18 h. The number of experiments (*N*) and the number of tumor‐cell islands (*N*) are indicated on each graph. For example, *N* = 3 and *N* = 97 indicate three repeats and a total number of 97 islands. g) The average tumor‐cell area and average cell number for tumor cells, in the presence and absence of UTD or CAR T cells (****p < 0.0001, one‐way ANOVA analysis). h) Percentage of surviving tumor cells at 18 h alone, with UTD T cells, and with four CAR T cells derived from four different healthy donors (****p < 0.0001, one‐way ANOVA analysis, *N* indicates the number of tumor‐cell islands measured). *N* indicates the number of islands.

We designed a second‐generation anti‐BCMA chimeric antigen receptor consisting of a single chain variable fragment (scFv) connected with a CD8 hinge/transmembrane domain to 4‐1BB and CD3ζ intracellular domains (**Figure** **2**a–d). In order to facilitate the evaluation of transduction efficiency with the lentiviral construct, we incorporated the mCherry fluorescent reporter gene after a T2A element at the C‐terminal of the CAR sequence (Figure 2a,b). Using flow cytometry, we determined that the efficiency of gene transfer into primary human T cells was 40–50% (Figure 2b). We also confirmed high and uniform expression of BCMA antigen by the multiple myeloma (MM) cell line RPMI 8226 by flow cytometry analysis (Figure 2c). To visualize and distinguish tumor cells from effector CAR T cells (mCherry positive), we engineered the tumor cells to express the green fluorescent protein (GFP).

A microscopic end‐point snapshot of MiTA enables a quantitative evaluation of the overall antitumor efficacy of CAR T cells. We observed a significant shrinkage of the BCMA+ RPMI 8226 tumor spots at 18 h with the presence of anti‐BCMA CAR T cells (Figure 2e). We quantified the efficacy of tumor‐cell elimination and found that the number of tumor cells decreased approximately fivefold, from an average of 77 cells per spot at *t* = 0 h down to an average of 15 per spot at *t* = 18 h (Figure 2f–h). At the same time, the area of tumor‐cell spot shrunk approximately sevenfold at 18 h (from 2.3 × 10^4^ at 0 h down to 3.2 × 10^3^ µm^2^ per spot at 18 h). In control experiments, untransduced (UTD) T cells were not able to eliminate tumor cells (Figure 2e). The number of surviving tumor cells were 3.9 × and higher than in the presence of CAR T cells and the tumor area 5.6 × higher (Figure 2g). The much lower percentage of tumor cells survived after 18 h with CAR T cells than UTD T cells confirms the efficient tumor killing by CAR T cells (17.1–39.3% vs 81.0% live tumor cells at 18 h, CAR T cells vs UTD) (Figure 2h). We also compared CAR T cells from four different healthy donors and found that substantial differences in antitumor activity, ranging from 17.1% (donor 3) to 39.3% (donor 2) (Figure 2h), despite a similar ≈50% transduction efficiency. Taken together, the end‐point snapshots of MiTA unveiled that CAR T cells eliminated the tumor cells more efficiently and consistently than the unspecific killing by UTD T cells and the antitumor efficacy varied among donors.

### 2.3 Dynamic Profiling of the CAR T Antitumor Activity Using MiTA

**Figure 3:**
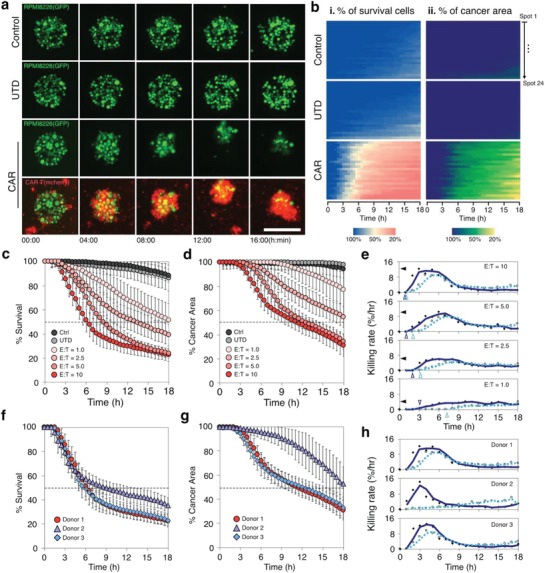
Dynamic profiling of the CAR T antitumor activity using MiTA. a) Fluorescent microscopic time‐lapse images show the morphological changes of tumor‐cell islands without T cells (control), with (UTD) T cells, or with CAR T cells, from 0 to 16 h. The scale bar is 200 µm. b) An array of heatmaps showing the progress of tumor‐cell elimination measured by percentage of surviving tumor cells and the remaining area of the tumor over 18 h, with CAR T cells, with UTD T cells, and without T cells. Each heatmap consists of data from 24 individual spots. The dark blue, yellow, and red indicate 100%, 50%, and 20% of surviving tumor cells correspondingly. The dark purple, green, and red indicate 100%, 50%, and 20% of remaining area of tumor. c) Average percentage of survival tumor cells and d) average percentage of tumor area over time at various E:T (*N* = 24 islands per condition). e) Killing rate over time at various E:T calculated from (c) and (d). The dark blue line and light blue dashed line represent the moving average (subsize = 2) of the killing rate calculated by the % survival tumor cells and % remaining tumor area correspondingly. The dark and light blue arrows indicate the starting point of elimination of individual tumor cells and shrinkage of tumor spots correspondingly. The black arrows indicate the peak killing rates. f) Percentage of survival tumor cells and g) percentage of remaining tumor area over time with CAR T cells transduced from three different donors (*N* = 24 islands per donor). h) Killing rate calculated from (f, dark blue line) and (g, light blue dashed line) over time for different donors.

The micropatterned tumor array enables us to visualize and quantify the dynamic process of tumor elimination by CAR T cells (**Figure** **3** and Video S1, Supporting Information). We distinguished two phases during the interaction between CAR T and tumor cells: an initial phase of CAR T cell accumulation at the tumor islands followed by a phase of rapid tumor killing. During the initial phase, which lasts ≈2 h, CAR T cells migrate toward the tumor islands (Figure 3a, CAR). Most tumor cells stay alive (Figure 3b‐i, CAR, blue region) and the initial morphology of the islands is retained (Figure 3b‐ii, CAR, purple region). During the second phase, lasting up to 18 h, CAR T cells eliminate tumor cells. During 3–6 h, the killing progresses rapidly, with a peak killing rate at ≈10% cells h^−1^ at effector to target cell ratio E:T = 10 (Figure 3a–c, CAR; Figure 3e, dark blue line, 3–6 h).

The killing of individual tumor cells induces rapid shrinkage of tumor spots (Figure 3d,e, blue dashed line). When ≈60% tumor cells were killed, the area of tumor decreased to < 40% (Figure 3c,d, E:T = 10). After 6 h, both the killing of individual tumor cells and the shrinkage of the tumor area slow down, with the killing rates gradually decreases to ≈2% h^−1^ (Figure 3e). The tumor cells are often lifted from the spots and carried around by the CAR T cell clusters (Figure 3a, CAR, 8–18 h). At 18 h, 75% tumor cells are eliminated and the tumor area decreases by 70% (Figure 3c,d). In contrast, UTD cells failed to kill tumor cells throughout the 18 h. Most tumor cells area alive, and the tumor islands retain their initial morphology (Figure 3a–d, UTD).

The dynamic profile of tumor‐cell elimination varies with the ratio of effector‐to‐target cells in two major aspects (Figure 3b–d). Loading more CAR T cells induces an earlier onset of tumor killing and tumor shrinkage. The killing of tumor cells started at 1, 1, 2, and 3 h and the decrease in the tumor area started at 1, 2, 3, and 7 h at E:T = 10, 5, 2.5, and 1, respectively (Figure 3e, dark and light blue hollow arrows). It is worth noting that the time difference between the onsets of tumor killing and island shrinkage also varies with E:T. At E:T = 10, the killing and shrinkage occur simultaneously. At E:T = 1, the shrinkage of tumor islands follows the killing, 4 h later (Figure 3e). Loading more CAR T cells accelerates the elimination of tumor cells and the shrinkage of the tumor area. The peak rate of killing decreases from ≈12% h^−1^ to ≈4% as E:T decreases from 10 to 1 (Figure 3e, black arrows) and the time to eliminate 50% tumor cells increases from 6 to 18 h (Figure 3c, black dashed line). The time to shrink the tumor area by 50% increases from 10 to 12 as the E:T decreases from 10 to 5. For smaller E:T ratios of 2.5 and 1, the tumor area remains > 50% at 18 h (Figure 3d, black dashed line).

### 2.4 CAR T Trafficking toward Tumor Spots

**Figure 4:**
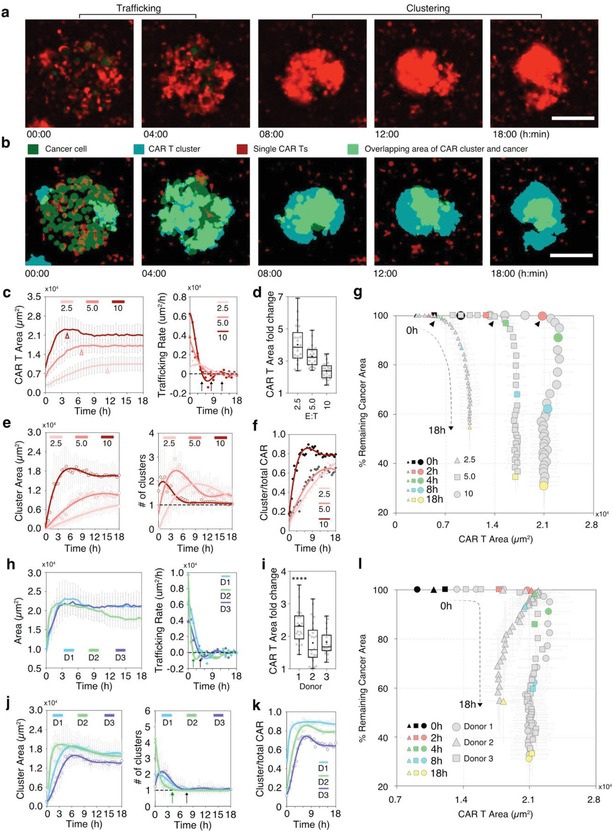
CAR T cell trafficking and clustering promote tumor‐cell killing. Panels (c)–(g) and (h)–(l) display readouts grouped by c–g) different E:T ratios or h–l) different donors. a) Time‐lapse fluorescent microscopic images demonstrate the trafficking of CAR T cells, the formation of CAR‐T cluster, and the shrinkage of the CAR‐T cluster on top of the tumor cells over time. The scale bar is 200 µm. b) Time‐lapse images highlighting the gradual clustering of CAR T cells and wrapping of tumor cells. The scale bar is 200 µm. c) The area of CAR T cells on the spot over time (left panel) and the calculated trafficking rate (right panel) at E:T = 2.5, 5, and 10 (*N* = 24 islands per condition). The red arrows indicate the plateau of trafficking. The black arrows indicate the time when the trafficking rate decreases to 0. d) Fold change of CAR T cell area at 18 h over 0 h at the 3 E:Ts. e) The area of CAR T clusters (left) and the corresponding number (right) of clusters on the spots (area > 1000 µm^2^) over time at the 3 E:Ts (*N* = 24 islands per condition). f) The ratio of CAR T cluster area to total area over time (*N* = 24 islands per condition). g) A graph showing the correlation between the trafficking of CAR T cells and shrinking of tumor cells during 18 h at the 3 E:Ts. 0, 2, 4, 8, and 18 h time points are highlighted with colored dots. The black arrows indicate the starting time of tumor‐cell killing. The density of dots along the X and Y directions reflects the rate of CAR T trafficking and the rate of tumor shrinkage correspondingly (*N* = 24). h) The area of CAR T cells on the spot over time (left panel) and the calculated trafficking rate (right panel) for three donors (*N* = 24 islands per donor). The arrows indicate the time when the trafficking rate decreases to 0 for donor 1, 3 (black) and 2 (green). i) Fold change of CAR T cell area at 18 h over 0 h for the three donors. j) The area of CAR T clusters (left) and the corresponding number (right) of clusters over time for the three donors. k) The ratio of CAR T cluster area to total area over time (*N* = 24 islands per donor). l) The correlation between the trafficking of CAR T cells and shrinking of tumor cells for the three donors.

We quantified the trafficking of CAR T cells at various cell densities (**Figure** **4**). We found that a rapid, initial trafficking phase is usually followed by a slow, plateau phase. The duration of the first phase depends on the E:T ratio. At E:T = 10 (high CAR T density), CAR T cells continuously migrated to the spots in the first 4 h with the CAR T cell area increased by threefold (Figure 4c). After 4 h, the trafficking plateaus and the accumulation rate decreases to ≈0 µm^2^ h^−1^ (Figure 4c right panel, black arrow). At lower E:T ratios, the trafficking is slower and the plateaus are delayed (Figure 4c right panel, 4, 6, and 12 h at E:T = 10, 5, and 2.5). The fold change in the CAR T area is larger at smaller E:T ratios. At 18 h, the area is increased by 4.0 ×, 3.2 ×, and 2.4 × at E:T = 2.5, 5, and 10, respectively (Figure 4d), confirming the robustness of trafficking.

We mapped the dynamic correlation between CAR T cell trafficking and the killing of tumor cells (Figure 4g). We found that at higher cell density (E:T = 5 and 10), CAR T cells exert efficient killing after the trafficking has plateaued. For example, in the first 2 h, while the CAR T area increases 3 ×, the area of tumor cells does not change (Figure 4g, E:T = 10, circles). From 2 to 8 h, the CAR T trafficking reaches the plateau and the area of tumor cells shrinks rapidly to 60% and 30% at 8 and 18 h, respectively.

### 2.5 CAR T Clusters Enhance Tumor‐Cell Killing

We observed that CAR T cells often form large clusters on top of target islands when killing the tumor cells (Figure 4a,b). During tumor‐cell killing, CAR T cells first formed multiple small clusters on top of the tumor island and then merge into one large cell cluster that engulfs the tumor cells within it (Figure 4b). In the first 6 h, the average cluster area increases from ≈1600 to 18 000 µm^2^ while the average number of clusters decreasing from 2 to 1, indicating the multiple small clusters merge into one large cluster (Figure 4e, E:T = 10). At 18 h, 80% of the CAR T cells merged into a single cluster on the spot (Figure 4f, E:T = 10) and tumor cells were enveloped within the CAR cluster completely (Figure 4b). At E:T ratios below 5, the clustering is slower and CAR T cells end up forming multiple smaller clusters (Figure 4e,f, E:T = 2.5 and 5). At 18 h, only 60% of CAR T cells formed clusters on the tumor island with the rest dispersed around tumor islands (Figure 4f, E:T = 2.5 and 5). The CAR‐T cluster to tumor area ratio increases to > 2 during 18 h, indicating effective control of the tumor by CAR T (Figure S2, Supporting Information). At low CAR T density (E:T = 2.5), the trafficking is slower and overlaps with the killing (Figure 4g, triangle). The killing is inefficient, and the CAR T clusters do not grow larger than the tumor‐cell area even at 18 h (Figure S2, Supporting Information).

### 2.6 Heterogeneity in Antitumor Activity of CAR T‐Cells from Different Donors

We found distinct dynamic profiles of tumor killing by CAR T cells originating from different healthy donors (Figure 3f–h). Tumor cells were killed equally efficient by CAR T cells from donor 1 and 3. However, tumor cells were killed less efficiently by CAR T cells donor 2 and the shrinkage of tumor islands was slower and delayed (Figure 3f–g, donor 2). Interestingly, tumor cells were killed faster during the first 3 h by the CAR T cells from donor 2, with the highest killing rate of ≈16% h^−1^ at 3 h (Figure 3h, donor 2). However, the killing slowed down after 3 h, with the rate sharply decreasing to ≈4% at 5 h and then ≈2% at 8 h. As a result, only 60% tumor cells were eliminated at 18 h (Figure 3g). The rate of area shrinkage remains < 4% throughout 18 h distinct from the other two donors (Figure 3h, light blue dashed lines).

We found that the CAR T cells from different donors also displayed different trafficking and clustering profiles (Figure 4h–j). CAR T cells from donor 1 exhibited the strongest trafficking with 2.5 × increase in the area after 18 h, much higher than donor 2 and 3 (Figure 4i). In addition, they showed the best ability to cluster around the tumor cells, with ≈90% cells merging in to a single cluster at 18 h (Figure 4k, blue line). The trafficking of CAR T cells from donor 2 was the fastest but plateaued the earliest among the three donors (Figure 4h, green line). Uniquely, the CAR T area kept decreasing after 3 h, leading to the smallest area at 18 h (Figure 4h). CAR T cells from donor 2 clustered the fastest and formed a single cluster at 4 h, earlier than the other two donors (Figure 4j, green lines and green arrow). CAR T cells from donor 3 exhibited a similar trafficking profile as donor 1 (Figure 4h,j, purple and blue lines). However, their ability to form clusters is weaker than the other two donors with only 60% of cells clustering together at 18 h (Figure 4k, purple). Mapping CAR T trafficking and tumor‐cell killing together, we found that the killing efficiency may relate to the area of CAR T cells on the spot (Figure 4l). Despite fast trafficking at the first 2 h (Figure 4l, red dots), the area of CAR T cells from donor 2 is smaller after 4 h than the other two donors. Correspondingly, the killing is less efficient, shown as a higher % of remaining tumor area at 4, 8, and 18 h (Figure 4l green, blue, and yellow triangles vs circles and squares).

### 2.7 Comparing the Antitumor Activity of Two CAR T‐Cell Constructs Using MiTA

We employed MiTA to compare the antitumor activity of anti‐BCMA and APRIL‐based CAR T cells toward BCMA positive and negative multiple myeloma MM.1s tumor cells. “A proliferation‐inducing ligand” (APRIL) is a soluble ligand that can bind BCMA and the transmembrane activator and calcium‐modulator and cyclophilin ligand (TACI), two antigens highly expressed on MM cells. We generated an APRIL‐based CAR consisting of a truncated APRIL fused to a spacer domain and to the same endodomain used for anti‐BCMA CAR construct (anti‐BCAR). Our hypothesis is that dual antigen targeting will enhance the tumor‐cell killing, reduce the incidence of antigen negative escape, and overall therapeutic potential.^22^

**Figure 5:**
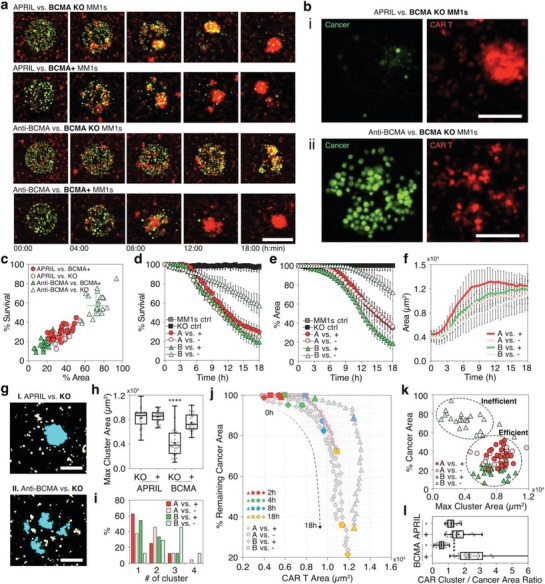
Comparing the antitumor activity of two CAR T‐cell constructs using MiTA. a) Time‐lapse fluorescent images demonstrating the killing of BCMA ± MM.1s by APRIL and anti‐BCMA CAR T cells during 18 h. The scale bar is 200 µm. CAR T cells and tumor cells are pseudocolored red and green correspondingly. (b) Zoom‐in images showing i) the efficient elimination of tumor cells and clustering of CAR T cells and ii) inefficient killing and scattered CAR T cells at 18 h. c) Quantification of surviving tumor cells and remaining tumor area at 18 h for the four combinations of CAR constructs and tumor‐cell types (*N* = 24 islands per condition). Each dot represents one island. Percentage of d) survival tumor cells and e) remaining tumor area over time. f) Trafficking of CAR T cells to the tumor island over time (*N* = 24 islands per condition). g) Microscopic images (post‐thresholding) showing clusters of CAR T cells (area > 1000 µm^2^) on a tumor spot at 18 h. The cyan regions represent the CAR T clusters. The yellow dots represent scattered CAR T cells. The scale bar is 100 µm. h) The average area of the largest cluster over 24 spots at 18 h. i) The probability of spots with 1–4 CAR T clusters for the four conditions. j) The correlation between the trafficking of CART cells and the shrinkage of tumor area during 18 h for the four conditions. The red line highlights the distinct profile of anti‐BCMA CAR versus BCMA‐tumor cells (B vs −). k) The CAR T cluster area versus the percentage of remaining tumor area at18 h. l) The ratio of CAR cluster to the tumor island areas at 18 h (****p < 0.0001, one‐way ANOVA analysis).

Our assay shows that APRIL‐based T cells can efficiently eliminate both BCMA positive and negative MM.1s tumor cells (**Figure** **5**a,b‐i,c–e). At 18 h, < 30% tumor cells survived the killing and area of the tumor decreased to ≈35% (Figure 5c). Anti‐BCAR T cells eliminate BCMA positive tumor cells more efficiently than APRIL‐based CAR T cells (19% surviving tumor cells and 19% remaining area at 18 h), however exhibited significant deficiency in killing BCMA negative tumor cells (Figure 5a,b‐ii,c–e). At 18 h, 57% tumor cells had survived the killing and the tumor area had only decreased to 72% (Figure 5c).

We assessed the dynamic profiles of tumor‐cell killing for both CAR T constructs (Figure 5d,e). APRIL‐based CAR T cells exhibited a similar profile for killing BCMA positive and negative MM.1s tumor cells (Figure 5d,e, dark and light dotted curves) with an interaction phase from 0 to 3 h and rapid elimination phase after 3 h. Anti‐BCAR T cells killed BCMA positive tumor cells more efficiently than APRIL‐based CAR T cells, which caused immediate and faster shrinkage of tumor area (Figure 5e). However, they exhibited deficient killing of BCMA negative tumor cells. The killing started immediately in the absence of the initial interaction stage seen in other conditions (Figure 5d). The killing rate was slower and the shrinkage of tumor occurred after 9 h, ≈5 h later than other conditions (Figure 5e).

We observed different trafficking dynamics for CAR T cells with different constructs (Figure 5f). The trafficking of APRIL‐based CAR T cells toward BCMA positive tumor spots is the fastest, leading to the largest CAR T area at 9 h among the four conditions (Figure 5f, dark red curve). After 9 h, the trafficking plateaus. The trafficking of APRIL‐based CAR T cells toward BCMA negative tumor cells and anti‐BCAR T cells toward BCMA positive tumor cells is slower and plateaus later than 9 h (Figure 5f, light red and dark green curves). Finally, the trafficking of anti‐BCAR T cells toward BCMA negative tumor cells was the slowest, progressing in a linear fashion from 3 to 18 h (Figure 5f, light green curve).

Although a similar number of CAR T cells arrive at the spots at 18 h (Figure 5f), the clustering of CAR T cells is different for the four conditions (Figure 5g–i). When CAR T cells form a single large cluster on the tumor spots, killing is efficient (Figure 5g‐I,h,i). When CAR T cells aggregate into multiple, small clusters, killing is inefficient (Anti‐BCAR vs BCMA negative tumor—Figure 5g‐II,h,i). The largest clusters on the spots in Anti‐BCAR versus BCMA negative tumors are significantly smaller than all other conditions (Figure 5h). During efficient killing, 37–63% of the spots have one single large CAR T cluster. In contrast, only 13% of spots have one single cluster in the anti‐BCAR CAR T versus BCMA negative tumor condition. Taken together, our results suggest that APRIL‐based CAR can form single, large clusters on both BCMA positive and negative tumor spots. However, anti‐BCMA cells failed to form clusters on BCMA negative tumors.

We mapped the correlation between the trafficking of CAR T cells and the corresponding killing which revealed distinct dynamic profiles during efficient and inefficient killing (Figure 5j–l). The results demonstrate distinct dynamics among the four conditions and highlighted the deficient killing of anti‐BCAR versus BCMA negative (Figure 5j, highlighted with a red line) and efficient killing in the other conditions. The clustering of CAR T cells also correlates with the killing efficiency (Figure 5k). Tumor cells were eliminated efficiently when CAR T cells formed large clusters (Figure 5k, efficient). In deficient killing situations, CAR T cells only form smaller clusters with a significantly smaller ratio of CAR cluster area to tumor area (Figure 5l).

In summary, MiTA enables high‐content analysis and multifaceted comparison of the antitumor activity between different CAR T constructs. Our data show that APRIL‐based CAR T cells can effectively migrate to tumor spots, form clusters, and eliminate both BCMA positive and negative tumor cells, while anti‐BCAR T cells failed to do so toward BCMA negative tumor cells. This result suggests that APRIL‐based CAR T cells could reduce the incidence of antigen‐negative escape and thus have stronger therapeutic potential.

## 3 Discussion

We developed a micropatterned tumor array (MiTA) that enables high‐content and dynamic profiling of the collective antitumor activity of CAR T cells against multiple myeloma tumor cells.^23^ Spatially patterning tumor cells into islands induces strong CAR‐T trafficking toward tumor targets of similar size and area and allows for simultaneous characterizations of the recruitment of effector cells and elimination of target cells. The microfluidic compartments minimize the mechanical perturbation acting on loosely adherent T cells and prevents artificial cell interactions induced by cell drifting. The integration of the microfluidic compartments in a multiwell plate format facilitates multiplexed and high‐throughput screening of CAR T cells which could expedite the testing of different tumor‐cell lines, CAR T constructs, and drug candidates.

Compared to widely used biochemical assays that only provide end‐point results, MiTA provides comprehensive information regarding CAR T cell trafficking and subsequent tumor killing. Compared to conventional cell‐based assays that probe the interactions of effector and target cells that are randomly distributed on a surface, MiTA enables monitoring of collective interactions of CAR T cells with spatially patterned tumor‐cell group, which revealed the potential impacts of CAR T cell recruitment and clustering on tumor cluster elimination. Compared to microfluidic and organ‐on‐a‐chip models, MiTA is more straightforward to setup and enables simultaneous characterizations of antitumor activities on a large number of structurally similar tumor islands, which may promote the robustness of screening.

The micropatterned tumor array exhibits high‐content information on the dynamic interaction between CAR T cells and tumor islands. The dimensionality of information can be expanded further to decipher this process with greater details. In addition to the area and number of cells and clusters, one could characterize shape factors such as aspect ratio, circularity, etc. as well as the correlation between the tumor and CAR T cluster shapes. Ultimately, multiple‐dimensional data may be introduced into a machine learning algorithm for better stratifying the efficiency of CAR T cells against tumor cells.

Studying the interaction between different CAR T cells and tumor‐cell types revealed a signature profile for the antigen‐specific killing of tumor cells. Efficient killing driven by antigen‐specific binding is characterized by an initial, slow accumulation phase and a subsequent rapid killing phase. In the initial phase, CAR T cells migrated from surrounding to the tumor‐cell island but exerted a limited cytolytic effect on the tumor cells. Later, the CAR T cells on the island merged into large clusters and exerted a strong cytolytic effect. In contrast, with inefficient antigen‐specific binding (anti‐BCAR vs BCMA negative MM.1s), the interaction lacks the initial phase and the killing is overall slower and less efficient.

The CAR T cell trafficking and clustering around tumor‐cell islands highlights the complex interactions involved in efficient killing of tumor cells. Our results confirm that trafficking is a robust phenomenon that is independent of CAR T density. Moreover, trafficking boosts the local ratio of effector to target cells on the niche, facilitating the killing of tumor cells. This finding echoes a recent in vivo observation in a mouse model of B cell lymphoma which showed that the density of CAR T cells increased by tenfold in 3 d in the bone marrow and the tumor clearance was correlated with the CAR T density.^24^ Together, our in vitro data and the in vivo model confirm the importance of CAR T trafficking in promoting tumor‐cell killing.

When exerting the cytolytic effect on a tumor island, CAR T cells merge into clusters around tumor cells and collectively shrink the tumor island. We found that the size and morphology of the CAR T cell clusters are correlated with the efficiency of clearance of the tumor cells on the island. The formation of a single large CAR T cluster on the island is always associated with better tumor clearance. The formation of multiple smaller clusters, either due to lower CAR T density or the absence of tumor antigen is related to deficient tumor clearance. These findings imply that efficient clustering of CAR T cells may play an important role in clearing tumor‐cell clusters. CAR T cell clustering has been recently reported in a mouse model of B cell lymphoma. CAR T cells formed large cell clusters around malignant B cells in the blood circulation 15 min after injection.^24^ Whether the cluster formation in vivo promotes tumor‐cell killing or follows the same dynamics observed in vitro remains to be investigated.

Our data show that APRIL‐based CAR T cells efficiently killed both BCMA positive and negative MM.1s, while anti‐BCMA CAR T cells failed to kill BCMA negative MM.1s. These in vitro data match the results from in vivo experiments which demonstrated that anti‐BCMA CAR T cells are unable to clear MM1.s BCMA KO cells engrafted in Non‐Obese Diabetic, Severe combined immunodeficient, Interleukin 2 gamma null (NOD‐SCID‐g chain/(NSG) mice) (manuscript under review). These results suggest that our platform could support the validation of CAR T cell efficacy. The trafficking and clustering of APRIL‐CAR T cells occurred in a similar fashion toward both tumor cells, while anti‐BCMA CAR T cells showed a deficient ability to form clusters on BCMA negative MM.1s. These suggest the potential of April‐CAR T cells to reduce the incidence of antigen‐negative escape, without compromising other key cell functions.

Although it permits multifaceted dynamic characterizations of CAR T cells with ease of use and high throughput, MiTA is not without limitations. For example, the recruitment of CAR T cells and their interactions with tumor cells happen on a 2D surface which may differ from those in a physiologically relevant 3D microenvironment. This limitation can be overcome by incorporating more features in MiTA. For example, dispensing CAR T cells embedded in hydrogel on top of tumor island array could realize the monitoring of CAR T cell recruitment and cytolytic activities in 3D. Co‐patterning tumor cells with other cellular components such as bone marrow stromal cells could provide a more sophisticated in‐vivo like microenvironment. Implementing more features in MiTA will shift it toward a more physiologically relevant model but complicate the preparation and the operation of the system at the same time. The versatility of MiTA platform allows possible system modification to adapt to the requirements of different studies and screenings.

The dynamic profiles of CAR T cell trafficking, clustering, and tumor elimination vary among healthy donors. The differences may stem from the intrinsic variations in T cell populations among donors or variations induced during CAR T cell manufacturing. The functions of CAR T cells among patients are likely to be poorer and vary even more. Deciphering the link between the variability and the corresponding clinical outcome will facilitate the production of more effective CAR T cells and could ultimately serve as a biomarker of response or a measure of T cell “fitness.”^25^ Ultimately, mapping the dynamic information from in vitro assays, multiomics data of patients, and the clinical outcome could create a landscape that aids the development of more efficient and personalized CAR T cell therapies.

## 4 Experimental Section

*Construction of CAR, T Cell Culture, and Transduction*: Anti‐BCMA and APRIL CAR constructs were synthesized and cloned into a third‐generation lentiviral plasmid backbone under the regulation of a human EF‐1α promoter. Anti‐BCMA CAR bears a CD8 hinge and transmembrane domain, 4‐1BB costimulatory domain, and CD3 zeta signaling domain. APRIL CAR bears a 4‐1BB transmembrane and costimulatory domains and CD3ζ signaling domain. Both vectors also contained a second transgene coding for the fluorescent reporter mCherry to facilitate enumeration of transduction efficiency. Human T cells were purified (Stem Cell Technologies, Catalog #15 061) from anonymous human healthy donor leukopacs purchased from the Massachusetts General Hospital (MGH) blood bank under an Institutional Review Board (IRB)‐exempt protocol. For primary T‐lymphocyte expansions, bulk human T‐cells were activated (day 0) using anti‐CD3/CD28 Dynabeads (LifeTechnologies), followed by transduction with a lentiviral vector encoding the CAR 24 h later as described.^26^ T cells were cultured in media supplemented with rhIL‐2 (20 IU mL^−1^) beginning on day 0 of culture and were maintained at a constant cell concentration (0.5 × 10^6^ mL^−1^) by counting every 2–3 d. T cells were de‐beaded at day 10 of culture and functional assays were performed at day 11, after resting overnight.

*Cell Lines and Culture Conditions*: Two B‐lymphoblast myeloma cell lines, RPMI 8226 and MM.1s, were purchased from American Type Culture Collection (ATCC). Cells were engineered to constitutively express click beetle green (CBG) luciferase/enhanced GFP (eGFP) and then sorted on a FACSAria (BD) to obtain a pure population (CBG‐GFP+) (≥99%). RPMI 8226 cells were cultured in RPMI media containing 10% fetal bovine serum (FBS), penicillin, and streptomycin. MM.1s cells were cultured in RPMI media supplemented with 20% FBS, penicillin, and streptomycin. MM.1s BCMA knockout cells were generated used CRISPR/Cas9 technology.

*Flow Cytometry*: Anti BCMA‐APC antibody was used to detect BCMA expression by flow cytometry (clone 19F2, BioLegend). Cells were stained for 30 min in the dark at 4 °C and washed twice in phosphate‐buffered saline with 2% FBS. 4′,6‐diamidino‐2‐phenylindole was added to gate in the viable cells before the acquisition. Samples were run on a Fortessa X‐20 (BD) and data analyzed with FlowJo (Version 10).

*Microspotting*: Poly‐L‐lysine solution at 0.1% (w/v) (Sigma‐Aldrich) and high‐molecular‐weight cationic ZETAG solution were mixed at a volume ratio of 100:1. The mixture was spiked with FITC‐tagged poly‐L‐lysine (Sigma‐Aldrich) for visualization. Using an automatic liquid dispenser (Picospotter, Poly‐Pico Technologies LTD), the solution was dispensed into 16 8 × 8 spot arrays on a 3 × 1 in. ultraclean glass slide (SuperChip Microarray Slides, Thermo‐Fisher Scientific). The spots were dried at room temperature for overnight. For optimal adhesion of cells on the spots, the slide should be used between15 and 48 h after spotting the material.

*Microfabrication of the Microchamber Membrane*: The PDMS membrane was fabricated with the standard soft lithography process. Briefly, a master mold was fabricated in a negative photoresist (SU‐8, Microchem) with a height of 300 µm on a 4 in. silicon wafer. PDMS base and curing agent (PDMS, Sylgard 184, Elsworth Adhesives) were mixed thoroughly at a ratio of 10:1 and cast on the wafer. To adapt membrane to the commercialized well frame, we fabricated the membrane with a thickness of 1.5 mm by casting PDMS mixture (13.5 g) on the 4 in. wafer. The wafer with the mixture was degassed in a vacuum chamber and then transferred to an oven (80 °C) to cure overnight. After curing, the membrane was diced and peeled from the wafer. Inlets and outlets were created at the four corners of each square chamber with a 1 mm diameter biopsy punch (Harris Uni‐Core). The membrane was then treated with oxygen plasma to hydrophilize the PDMS surface.

*Device Assembly and Operation*: The spotted slide and the PDMS membrane were manually aligned and assembled with a commercialized 16‐well chamber frame (ProPlate, Grace Bio‐Labs). Tumor‐cell sample (20 µL, 25million mL^−1^) were loaded in a microchamber with a pipette. After 5 min, cells that were not on the spots were removed by flushing the chamber with RPMI + 10% FBS (200 µL). After patterning the tumor cells, fibronectin solution (R&D systems) (20 µL, 10 µg mL^−1^) was loaded in the microchamber and incubated at 37 °C and 5% CO_2_ for 2 h. The microchamber was washed with media (100 µL) and then covered with media (200 µL) to prevent evaporation during the experiments. CAR T cell suspension (20 µL) at desired concentrations was loaded into the chamber. Finally, the 16‐well chamber frame was sealed with a transparent sticky film and was ready for the time‐lapse imaging.

*Time‐Lapse Microscopy*: Time‐lapse fluorescent microscopy was employed to image the migration and antitumor activity of CAR T cells. Images were taken at 100–300 locations using a 10× or 20× objective with a time interval of 15–30 min using a fully automated Nikon TiE microscope (Micro Device Instruments). The microscope is equipped with a heat chamber which provides 37 °C, 5% CO_2_ and humidity for long term imaging. Files in .nd2 format were imported into Fiji ImageJ for analysis.

*Image Processing and Data Analysis*: Time‐lapse images were processed in Fiji ImageJ. The area and roundness of the fluorescent poly‐L‐lysine spots were measured by setting an automatic threshold to the images, followed by Analyze Particles function. The number of tumor cells on a spot was measured automatically with Trackmate module in ImageJ. The “estimated cell sizes” were set to 14 and 10 µm and “intensity thresholds” to 3 and 0.5 for RPMI 8226 and MM1s tumor cells, respectively. These parameters were selected based on the cell size and GFP intensity of each cell line. It was verified that these parameters generate accurate cell counts by comparing the number counted automatically and manually. After confirming the accuracy, the parameters were fixed for all the experiments. The percentage of remaining tumor cells was calculated as the number of cells at a time point over the initial number of cells. The area of tumor cells and CAR T cells on a spot were measured in ImageJ using a macro. The macro set automatic thresholds to a stack of fluorescent images in either Triangle or Huang modes and measured the area of GFP (tumor cells) or mCherry (CAR T cells) positive objects in the image stack. The data were imported into Excel. The graphs were plotted using excel, R and GraphPad Prism.

To calculate the ratio of effector to target cells (E:T ratio), the area density of CAR T cells was calculated first according to the concentration of CAR‐T suspension and the dimensions of the chamber. Then, the number of CAR T cells in a 500 × 500 µm^2^ region was calculated. This area was chosen because the 64 tumor islands were arranged into an 8 × 8 array spaced 500 µm apart. The ratio of CAR‐T/tumor cells calculated the number of CAR T cells in the 500 × 500 µm^2^ region over the average number of tumor cells on a spot.

## Conflict of Interest

The authors declare no conflict of interest.

## Supporting information

Supplementary

Supplementary
