# Supplementary material for: Dynamic Profiling of Antitumor Activity of CAR T Cells Using Micropatterned Tumor Arrays
Source: Adv Sci (Weinh). 2019 Sep 30;6(23):1901829. doi: 10.1002/advs.201901829 (PMC6891905; doi:10.1002/advs.201901829)
Supplement: Supplementary file 1 — Supplementary [file ADVS-6-1901829-s002.pdf]

Copyright WILEY-VCH Verlag GmbH & Co. KGaA, 69469 Weinheim, Germany, 2019.

ADVANCED SCIENCE
Open Access

Supporting Information

for *Adv. Sci.,* DOI: 10.1002/advs.201901829

Dynamic Profiling of Antitumor Activity of CAR T Cells Using Micropatterned Tumor Arrays

*Xiao Wang, Irene Scarfò, Andrea Schmidts, Mehmet Toner, Marcela V. Maus, and Daniel Irimia**

Supplementary information

Dynamic Profiling of Antitumor Activity of CAR T Cells using Micropatterned Tumor Arrays

Xiao Wang, Irene Scarfò, Andrea Schmidts, Mehmet Toner, Marcela V. Maus and Daniel Irimia

Supplementary Table S1 A table comparing the features of 4 *in vitro* approaches for the pre-clinical screening of cancer immunotherapy

Supplementary Figure S1. Photographs showing the 64 well plate platform for high-throughput, multiplexed screening. The 64 well plate contains 4 slides (i). Each slide has 16 wells (ii). Each well contains 64 tumor islands (iii).

Supplementary Figure S2. The ratio of the total CAR T area (left) and the cluster area (right) to the tumor area over time at E:T ratio of 2.5, 5 and10 (N=24).

Supplementary video 1: A microscopic video showing the dynamic process of CAR T cells killing tumor cells on 4 individual tumor islands.

Supplementary Table S1 A table comparing the features of 4 *in vitro* approaches for pre-clinical screening of cancer immunotherapy

Features	This tool	Biochemical	Real-time monitoring + cell culture plate	Microfluidics / organ-on-a-chip
**End-point killing**	Yes	Yes	Yes	Yes
**Dynamic killing**	Yes	No	Yes	Yes
**Trafficking of effector cells**	Yes	No	No	Yes
**Ease of use**	Yes	Yes	Yes	No
**Ease of setup**	Yes	Yes	Yes	No
**Robustness**	Yes	Yes	Yes	No
**Physiological relevance**	No	No	No	Yes
**Throughput**	High	High	High	Low
**Preparation-to-answer time**	< 1 day	< 1 day	< 1 day	>3 days
**High-content**	Yes	no	no	Yes

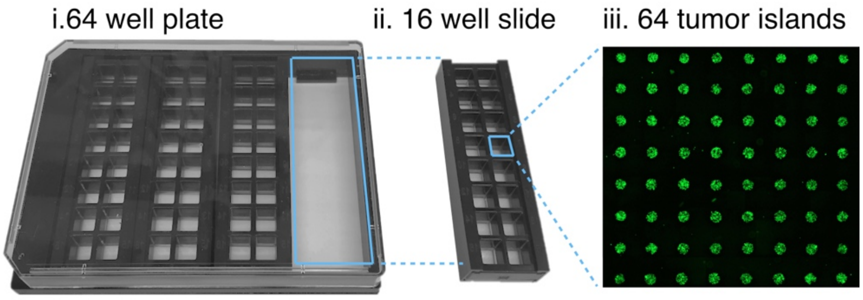

Supplementary Figure S1. Photographs showing the 64 well plate platform for high-throughput, multiplexed screening. The 64 well plate contains 4 slides (i). Each slide has 16 wells (ii). Each well contains 64 tumor islands (iii).

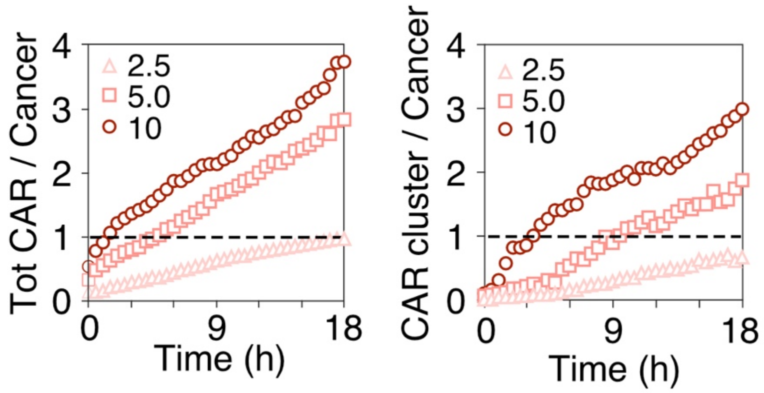

Supplementary Figure S2. The ratio of the total CAR T area (left) and the cluster area (right) to the tumor area over time at E:T ratio of 2.5, 5 and10 (N=24).
